# Changes in intracellular energetic and metabolite states due to increased galactolipid levels in *Synechococcus elongatus* PCC 7942

**DOI:** 10.1038/s41598-022-26760-4

**Published:** 2023-01-05

**Authors:** Kumiko Kondo, Rina Yoshimi, Egi Tritya Apdila, Ken-ichi Wakabayashi, Koichiro Awai, Toru Hisabori

**Affiliations:** 1 Laboratory for Chemistry and Life Science, Institute of Innovative Research, Tokyo Institute of Technology, Nagatsuta 4259-R1-8, Midori-Ku, Yokohama, 226-8503 Japan; 2 School of Life Science and Technology, Tokyo Institute of Technology, Nagatsuta 4259, Midori-Ku, Yokohama, 226-8503 Japan; 3 Department of Biological Science, Faculty of Science, Shizuoka University, Suruga-Ku, Shizuoka, 422-8529 Japan

**Keywords:** Microbiology, Plant sciences

## Abstract

The lipid composition of thylakoid membranes is conserved from cyanobacteria to green plants. However, the biosynthetic pathways of galactolipids, the major components of thylakoid membranes, are known to differ substantially between cyanobacteria and green plants. We previously reported on a transformant of the unicellular rod-shaped cyanobacterium *Synechococcus elongatus* PCC 7942, namely SeGPT, in which the synthesis pathways of the galactolipids monogalactosyldiacylglycerol and digalactosyldiacylglycerol are completely replaced by those of green plants. SeGPT exhibited increased galactolipid content and could grow photoautotrophically, but its growth rate was slower than that of wild-type *S. elongatus* PCC 7942. In the present study, we investigated pleiotropic effects that occur in SeGPT and determined how its increased lipid content affects cell proliferation. Microscopic observations revealed that cell division and thylakoid membrane development are impaired in SeGPT. Furthermore, physiological analyses indicated that the bioenergetic state of SeGPT is altered toward energy storage, as indicated by increased levels of intracellular ATP and glycogen. We hereby report that we have identified a new promising candidate as a platform for material production by modifying the lipid synthesis system in this way.

## Introduction

In recent years, photosynthetic microorganisms have attracted attention as a platform for material production that contributes sustainable development of our society. Among them, cyanobacteria are particularly attractive due to their established genetic background and genetic tractability, and various studies are being actively conducted to realize industrial applications using this organism^1–6^. In order for photosynthetic biomanufacturing by cyanobacteria to achieve significant success at the commercial level, challenges have been reported for metabolic engineering approaches to optimize carbon flux^7,8^, carbon dioxide fixation^9,10^, and improving photon capture^11^. In this study, we focus on the modification and enhancement of thylakoid membrane lipids, the site of photosynthesis, and we have finally succeeded in significantly increasing ATP and glycogen levels in the cell.

**Figure 1:**
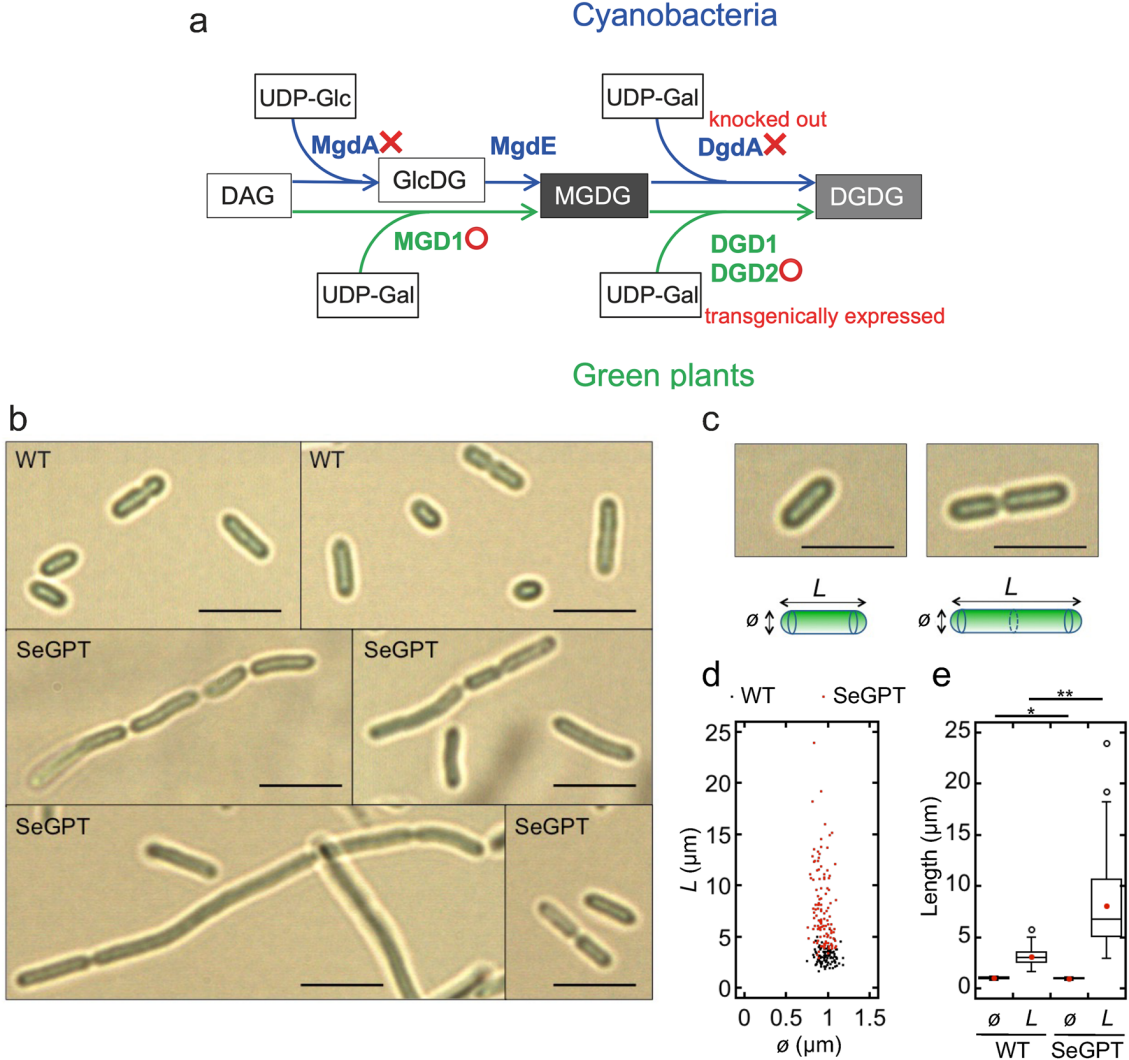
Schematic diagram of galactolipid-biosynthetic pathways in SeGPT (**a**), bright-field microscope images of *Synechococcus* (**b**) and measurements of cell size (**c–e**). (**a**) Lipid molecules and nucleotide sugar donors are depicted in boxes. The upper depicts a pathway in cyanobacteria and the lower depicts one in green plants. In SeGPT, two endogenous genes, encoding MgdA and DgdA, were knocked-out (red crosses) and two plant-type genes, encoding MGD1 from *Cucumis sativus* and DGD2 from *Arabidopsis thaliana* were transgenically expressed (red circles). DAG, diacylglycerol; GlcDG, monoglucosyl diacylglycerol; MGDG, monogalactosyldiacylglycerol; DGDG, digalactosyldiacylglycerol; UDP-Glc, uridine diphosphate-glucose; UDP-Gal, uridine diphosphate-galactose. Using the microscopic images of cells at the mid-log phase (**b**), cell length (*L*), and diameter (*ø*) were compared between the WT and SeGPT (**c**). Cell particles connected in a filamentous manner were counted as one cell (**c**, right). Results are presented in dot plots (**d**) and box plots (**e**). Black and red dots indicate the WT and SeGPT, respectively (**d**). Red circles in **e** indicate the averages (n = 102 for the WT; n = 118 for SeGPT). Scale bars: 5 µm (**b**, **c**). The asterisks indicate statistical significance (*P < 0.001, **P < 0.1^25, Welch’s t-test).

The lipid composition of the thylakoid membrane is widely conserved among oxygenic photosynthetic organisms. Two galactolipids, monogalactosyldiacylglycerol (MGDG) and digalactosyldiacylglycerol (DGDG), make up more than 70% of the lipid composition: 76% of the whole cell of *Synechocystis* sp. PCC 6803^12^ and 80% of the thylakoids of spinach chloroplasts^13^. This is a unique feature of the thylakoid membrane as phospholipids are the main component of other biomembranes. Several research groups have shown that the synthesis systems of these galactolipids in the chloroplasts of green plants and cyanobacteria are quite different (Fig. 1a). In cyanobacteria, MGDG is synthesized via a two-step reaction^14^. First, the enzyme MgdA uses uridine diphosphate (UDP)-glucose as a substrate and adds its glucose moiety to diacylglycerol to synthesize the intermediate monoglucosyl diacylglycerol (GlcDG)^15^. Next, the enzyme MgdE synthesizes MGDG via the isomerization of the glucose moiety of GlcDG^16^. In contrast, the enzyme MGD in green plants uses UDP-galactose as a substrate and directly synthesizes MGDG in a single reaction^17^. For the synthesis of DGDG from MGDG, the reaction is similar in cyanobacteria and green plants, but the origins of the enzymes involved, DGDG synthase (DgdA) for cyanobacteria and DGD for green plants, are quite different^18–20^.

Partial or complete disruption of the biosynthetic pathways of these lipids has been shown to have severe effects in some species. In *Arabidopsis*, a mutant in which monogalactosyldiacylglycerol synthase 1 (MGD1) protein levels are reduced by around half exhibits abnormal development of its thylakoid membranes and a chlorosis phenotype^21^; however, a complete knockout of *mgd1* disrupted the thylakoids, preventing the mutant from growing photoautotrophically^22^. These results indicate that galactolipids are important for the development of thylakoid membranes, or assembly of photosynthetic apparatus. To date, no cyanobacterial engineered or natural mutants with complete *mgdA* knockout have been reported^15,23^, suggesting that this gene plays a crucial role in cell survival. However, all of the aforementioned mutants showed a decrease in (or lack of) galactolipids, and it is not clear what would happen if the amount of galactolipids in the cell increased.

We reported previously on a transformant of the unicellular rod-shaped cyanobacterium *Synechococcus elongatus* PCC 7942, namely SeGPT, in which the MGDG and DGDG synthesis pathways were completely replaced by green plant–type enzymes^24^ (Fig. 1a). In SeGPT, the endogenous genes encoding MgdA and DgdA were knocked out by the insertion of antibiotic genes, and two genes for green plant–type galactolipid biosynthesis, namely *CsMGD1* and *AtDGD2*, were introduced into the neutral loci with an overexpressing promoter. These modifications were segregated, and the resultant SeGPT cells could grow photoautotrophically, indicating that cyanobacterial galactolipid biosynthesis pathways can be functionally complemented by the corresponding green plant–type pathway. In the mutant cells, the content of DGDG was significantly increased compared with that in the wild-type (WT) cells, and the total lipid content in the mutant was 1.7-fold higher than that in the WT. The cell proliferation rate was retarded in SeGPT under normal growth conditions; however, there was no such difference in the oxygen-evolving activity^24^. In this paper we examined how the increase in lipid content causes cellular effects in order to verify the possibility that this SeGPT strain could serve as a platform for photosynthetic material production.

Microscopic observations revealed abnormal cell division and heterogeneous distribution of thylakoids. We also found that intracellular ATP and glycogen levels were excessively accumulated in SeGPT, although the detailed molecular mechanisms are unknown. These results indicate that displacement of the galactolipid biosynthetic pathway has pleiotropic effects in *Synechococcus* cells and changes the bioenergetic state of SeGPT, making it a good candidate for a platform for the production of useful substances.

## Results

### Cell morphology and thylakoid development of SeGPT

**Figure 2:**
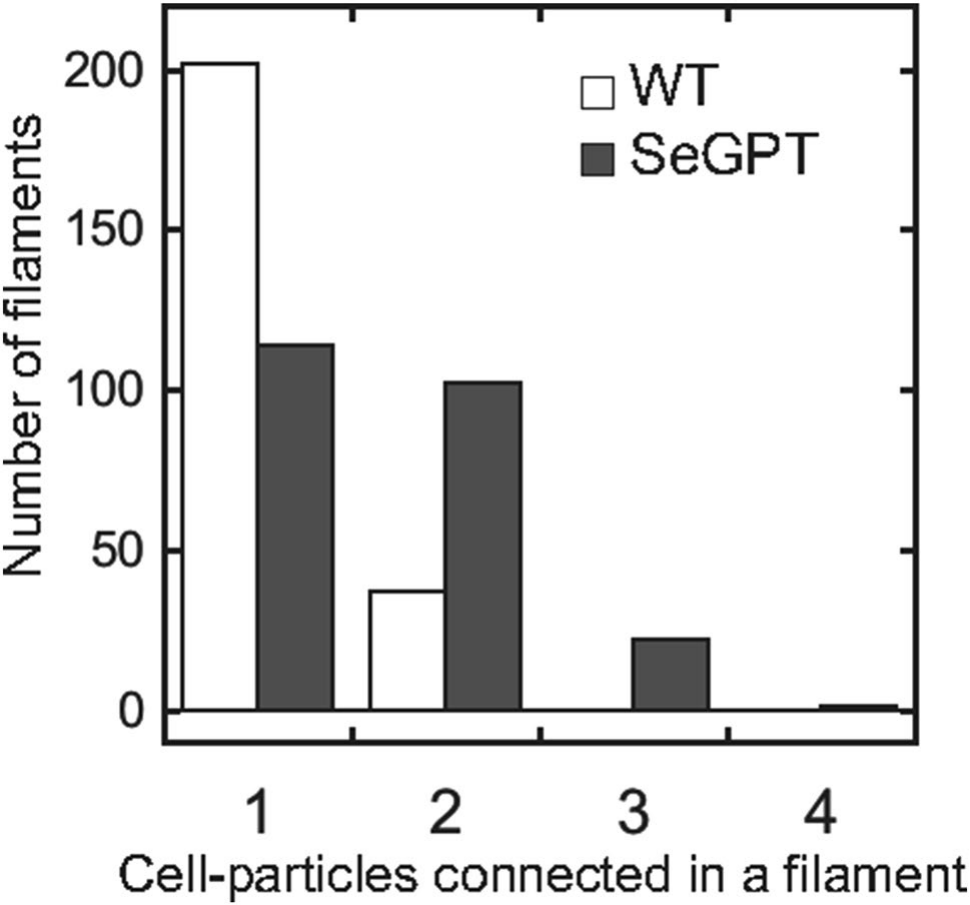
Number of connected cell particles within a filament. Bar graphs shows the number of connected particles (n = 239 for the WT and SeGPT).

In SeGPT cells, a marked increase in the levels of the thylakoid membrane–specific lipids MGDG and DGDG was previously observed^24^. To determine the influence of these effects on cell morphology, we first observed the transformant cells at the mid-log phase under an optical microscope, finding that the SeGPT cells were elongated (Fig. 1b). The cell length was significantly longer in SeGPT cells than that in WT cells (8.05 ± 3.79 and 3.09 ± 0.74 µm, respectively), but there was no significant difference in the diameter of these cells (0.93 ± 0.08 and 0.98 ± 0.09 µm, respectively) (Fig. 1c,d,e, Table 1). Similar observations were made at the early-log phase (Fig. S1). In SeGPT, three or more cells frequently remained connected in a filamentous manner (Fig. 1b), whereas only two cells were connected at most in the WT, which had a lower percentage of filamentous cells than that in SeGPT (Fig. 2). These results suggest that abnormal cell division occurs in SeGPT cells.

**Table 1:** Estimation of cell volume and evaluation of cellular compounds per cell-occupied volume.

	WT	SeGPT
Diameter (*ø*, µm)	0.98 ± 0.09 (n = 102)	0.93 ± 0.08 (n = 118)
Length (*L*, µm)	3.09 ± 0.74 (n = 102)	8.05 ± 3.79 (n = 118)
Cell volume (µm^3^)	2.07	5.30
Cell number (× 10^8^ OD_750_^−1^)	3.04 ± 0.39 (n = 12)	1.70 ± 0.34 (n = 12)
Cell-occupied volume ‰ (OD_750_ = 1)	0.63	0.90
Chlorophyll content (µg ml^−1^ OD_750_^−1^)	7.94 ± 0.29 (n = 4)	7.53 ± 0.31 (n = 4)
**Ratio to WT per cell volume**		
Chlorophyll	–	1.35
Total lipids^a^	–	1.16
F_o_F_1_-β	–	2.54
ATP	–	1.40
Glycogen	–	4.28

^a^Data for total lipid content are based on Apdila et al.^24^.

**Figure 3:**
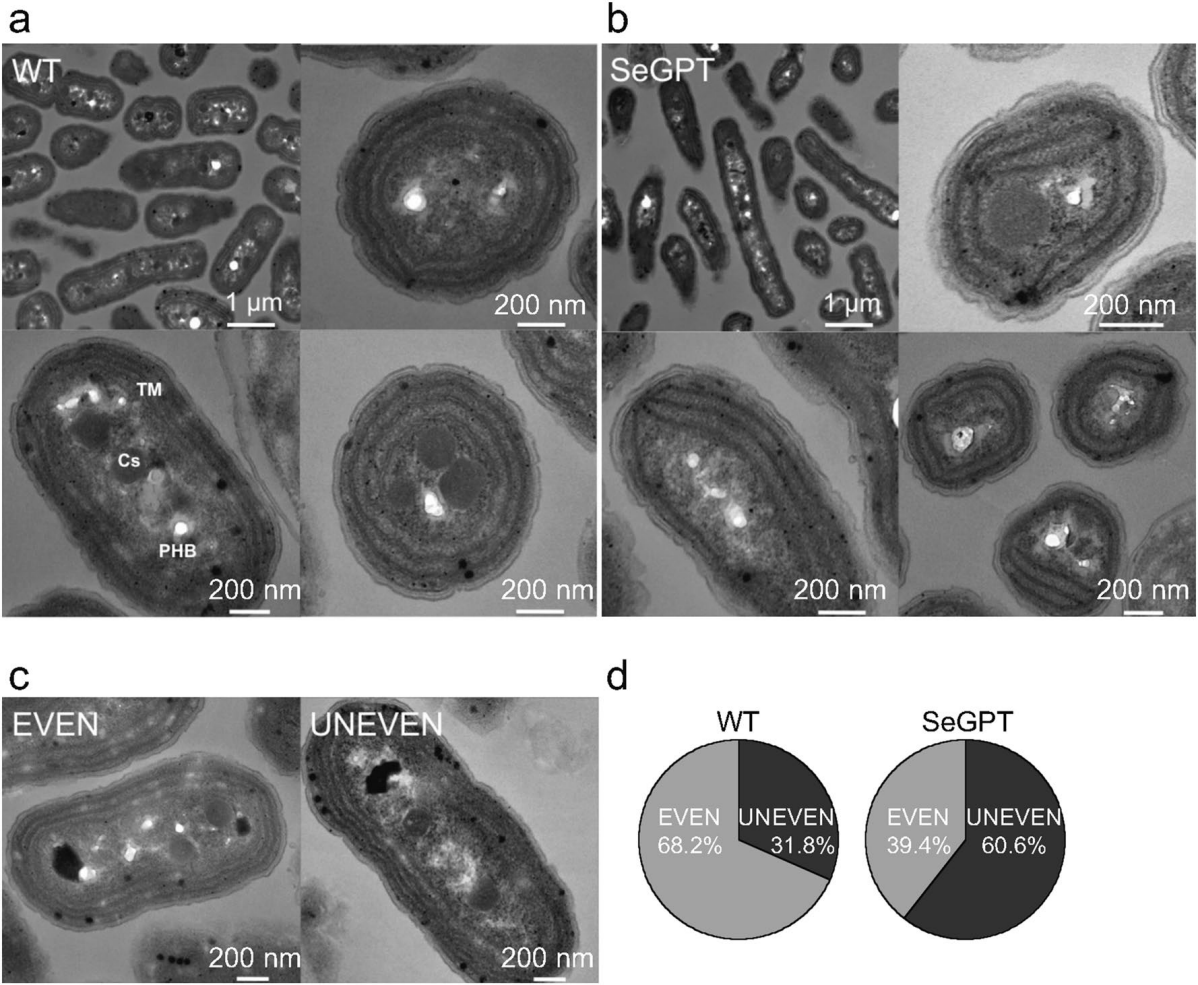
Transmission electron microscopic images of the WT (**a**, **c**) and SeGPT. (**b**) Carboxysomes (Cs), polyhydroxybutyrate (PHB), and thylakoid membrane (TM) (**a**). (**c**) The representative images of cells in which the thylakoid membrane is evenly developed in the cell (EVEN) and those in which it is unevenly developed (UNEVEN). (**d**) The percentages of EVEN and UNEVEN cells are compared between WT and SeGPT. Scale bars: 200 nm or 1 µm.

The increased levels of MGDG and DGDG in SeGPT cells^24^ suggest that the formation of the thylakoid membrane is denser than that in the WT, i.e., the number of thylakoid layers increases significantly. Therefore, we observed the cells under an ultrathin-section transmission electron microscope. The thylakoid membranes appeared to be formed in layers in both SeGPT and WT cells (Fig. 3a,b). As shown in Fig. 3c, cells with homogeneously distributed thylakoid membranes were designated as “EVEN,” whereas cells with heterogeneously distributed thylakoid membranes (e.g., showing a difference in the number of thylakoid layers between the left- and right-hand sides of the thin section of cells) were designated as “UNEVEN.” The percentages of EVEN and UNEVEN cells were compared between the WT and SeGPT, with the percentage of UNEVEN cells found to be 60.6% in SeGPT and 31.8% in the WT (Fig. 3d), indicating that the intracellular distribution of thylakoid membranes in SeGPT is more heterogeneous than that in the WT.

Notably, the morphology of the margins of the colonies on BG11 agar in plate cultures also differed in SeGPT compared with that in the WT (Fig. S2), suggesting that the composition of extracellular polysaccharides was affected in SeGPT. However, the cell surface layer could not be defined clearly in the electron microscope images (Fig. 3a,b), probably because the surface of the samples was damaged to some extent during the preparation procedures used prior to electron microscopy.

### Quantification of energy metabolites

**Figure 4:**
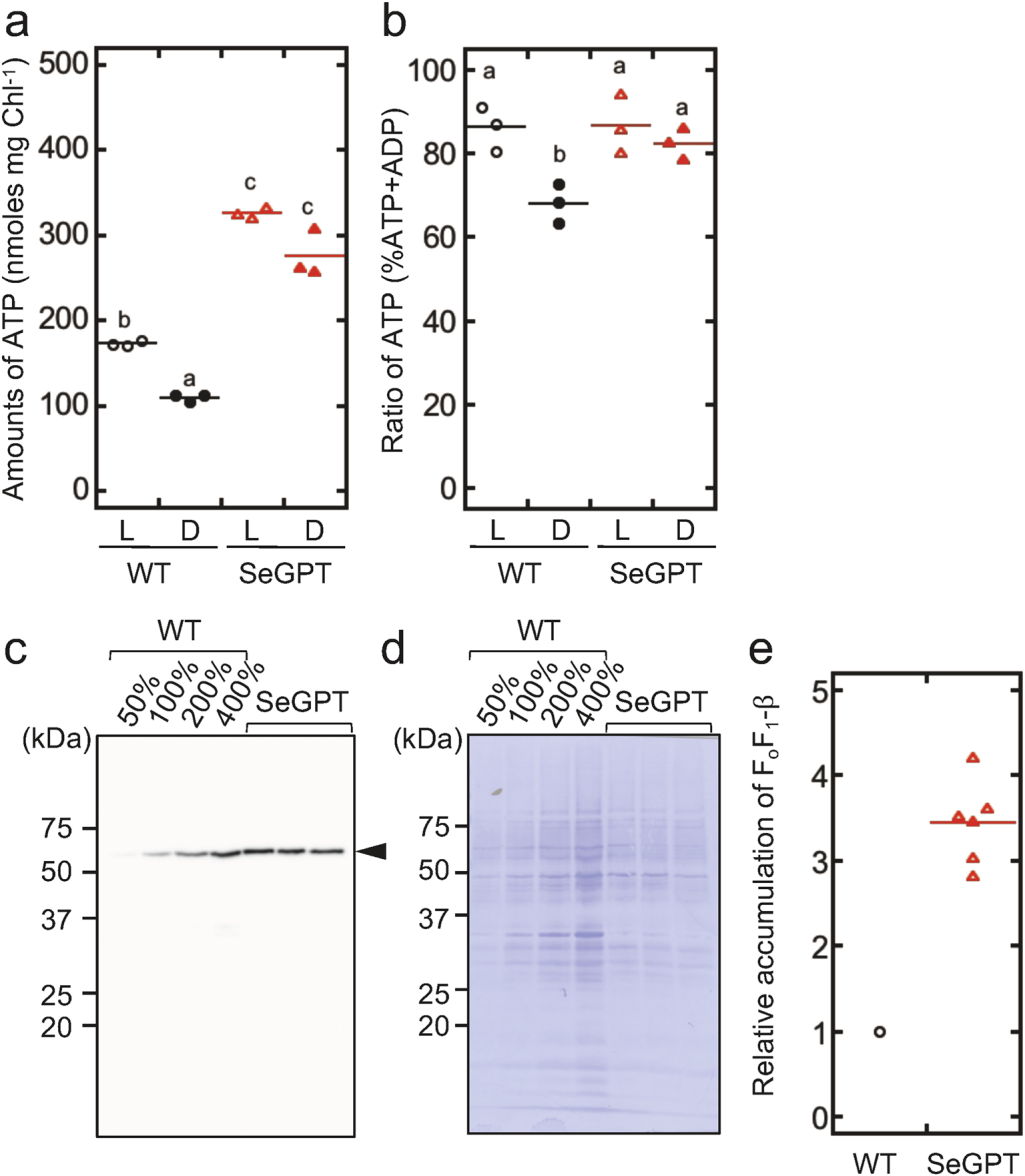
Measurements of intracellular ATP (and ADP) contents (**a**, **b**), and the quantification of F_o_F_1_-ATP synthase accumulation via immunoblot analysis using β-subunit antibodies (**c**–**e**). (**a**) Steady-state intracellular ATP content normalized to chlorophyll content. (**b**) The ratio of ATP/ATP + ADP. Cells were grown under continuous illumination (L), after which they were subjected to dark treatments for 15 min (D) and fixed with 2% (w/v) perchloric acid. After neutralization, ATP (or ATP + ADP) in the supernatant was quantified using a luciferin–luciferase assay. Different letters indicate significant differences (*p* < 0.05; Tukey–Kramer multiple comparison tests). For the quantification of ATP + ADP, prior to the luciferin–luciferase assay, neutralized aliquots were incubated for 3 h at 25 °C in the presence of pyruvate kinase. (**c**, **d**) Immunoblot analysis using the F_o_F_1_-β antibodies (**c**) and CBB-stained membrane (**d**) in the membrane fractions of the WT and SeGPT. Thylakoid membrane proteins equivalent to 0.75 µg chlorophyll were loaded for 100% WT and SeGPT. Three extracts from independent experiments were loaded for SeGPT. Full-length images are shown in Fig. S6. (**e**) Relative accumulation in SeGPT was calibrated according to that of the WT.

We next investigated the possibility that the aforementioned abnormal morphologies of the cell and the thylakoid formation affect cellular energy metabolism, as reported by Yamauchi et al.^25^, in which a mutation in a transcription factor alters the intracellular energy metabolism, resulting in cell gigantism. Figure 4a shows the quantification of intracellular ATP content at the stationary illuminated/dark phases. We reported previously that intracellular ATP content fluctuates substantially under light and dark conditions in the unicellular cyanobacterium *Synechocystis* sp. PCC 6803^26,27^. Similarly, in the present study, ATP levels in the WT *Synechococcus* cells showed oscillations in response to light and dark conditions (Fig. S3). Under light-illuminated conditions, the ATP content was 172 ± 12 and 325 ± 34 nmol mg chlorophyll^−1^ for the WT and SeGPT, respectively (Fig. 4a); thus, the ATP content in SeGPT was 1.9-fold higher than that in the WT. We quantified the total ATP and ADP content (ATP + ADP) under the light conditions and found that there was no significant difference in the ratio of ATP/ATP + ADP between the WT and SeGPT (86.2% ± 5.4% and 86.6% ± 7.0%, respectively) (Fig. 4b). After incubation in the dark for 15 min, the ATP/ATP + ADP ratio was reduced to 68.0% ± 4.7% in the WT but did not decrease substantially in SeGPT (82.4% ± 3.7%). As shown in Fig. S3, changes in the amount of ATP during the light-to-dark transition indicated that SeGPT showed a decrease in ATP levels similar to that of the WT in response to this transition; however, the range in SeGPT was lower than that in the WT. According to these results, we speculated that the accumulation of F_o_F_1_-ATP synthase (F_o_F_1_) might differ between SeGPT and the WT; thus, we quantified F_o_F_1_ accumulation. Immunoblotting analysis was conducted using antibodies against the β-subunit of F_o_F_1_ (F_o_F_1_-β; Fig. 4c,d). We quantified the chemiluminescent intensity of F_o_F_1_-β in SeGPT and determined its accumulation, which was estimated via calibration with the F_o_F_1_-β accumulation in the WT. We found that the accumulation of F_o_F_1_-β in SeGPT was 3.44 ± 0.49 times higher than that in the WT (Fig. 4e), suggesting that the F_o_F_1_ complex is significantly more abundant in SeGPT.

**Figure 5:**
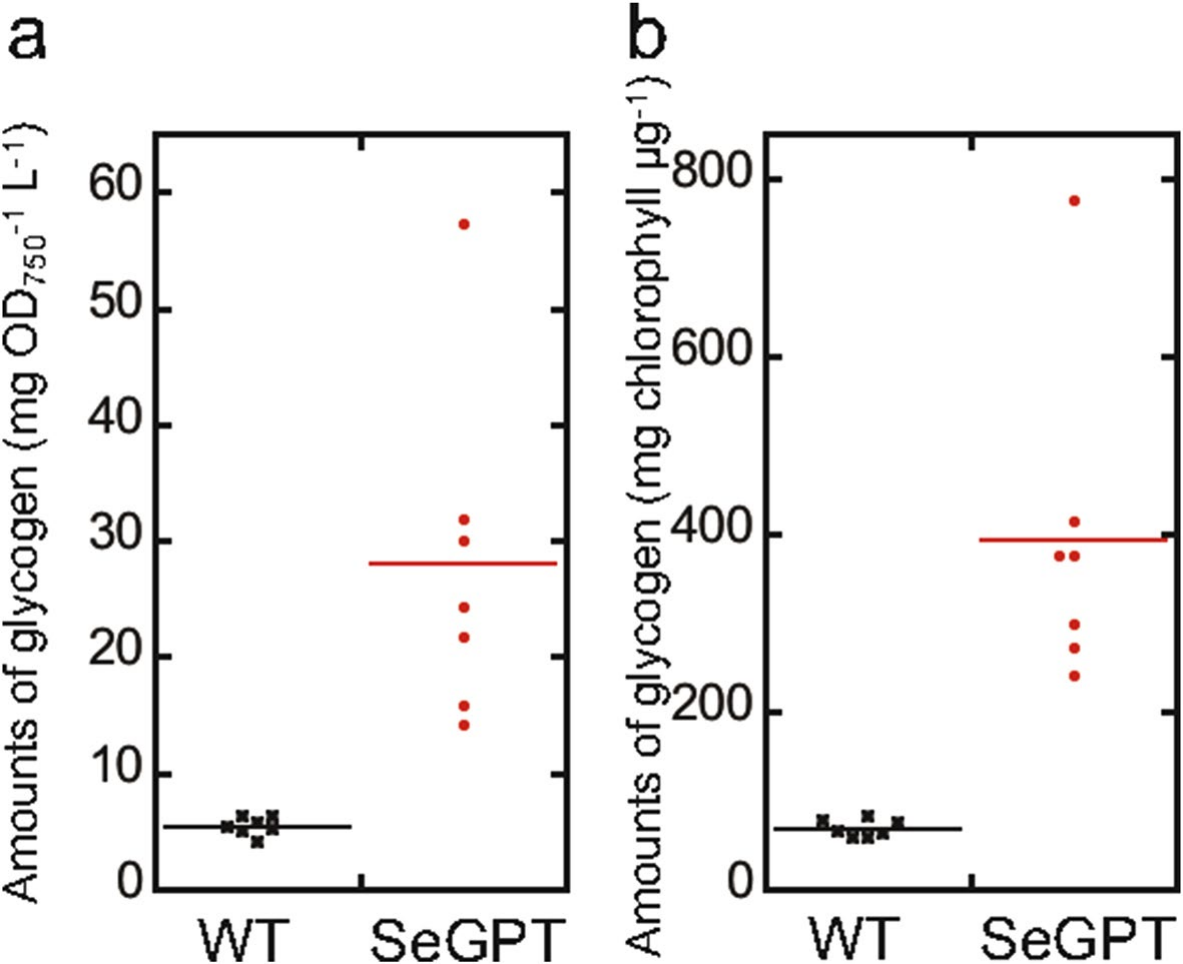
Measurements of intracellular glycogen content. WT and SeGPT cells grown under continuous light conditions were withdrawn at the indicated times and intracellular glycogen was quantified. Data were normalized to OD_750_ (**a**) or chlorophyll (**b**).

Interestingly, the ATP concentration under light and dark conditions remained relatively unchanged and at high levels in SeGPT (Figs. 4a, S3); thus, we speculated that sugar synthesis and/or accumulation might be abnormal in SeGPT. Therefore, we evaluated the energetical status of SeGPT cells by assessing their glycogen content (Fig. 5). Under continuous light conditions, the glycogen content in SeGPT was 5.2 times (normalized to OD_750_) or 5.8 times (normalized to chlorophyll) higher than that in the WT.

Although our data were normalized to either cell density, which is reflected in OD_750_, or chlorophyll content, the abnormal cell morphology in SeGPT cells (Fig. 1b) suggested that the number of cells per OD_750_ may also differ from that in the WT, which might complicate the interpretation of our results. Thus, assuming that the cells were cylinders with hemispheric caps at both ends (Fig. 1c), we quantified cell volume. Cell numbers and chlorophyll content per OD_750_ were also examined, and we calculated the concentration of each compound per volume occupied by the cell (Table 1). The amount of chlorophyll per OD_750_ differed slightly from that reported in our previous study^24^ presumably due to differences in culture conditions. Based on these analyses, we reached the same conclusion, i.e., that the levels of energy-related compounds, namely, F_o_F_1_-β, ATP, and glycogen increased in SeGPT, as did the level of total lipid content. These findings suggest that the energy status in SeGPT cells is altered from that in WT cells, as shown by the retardation in cell proliferation in the former^24^.

### Measurements of photosynthetic activity using chlorophyll fluorescence

**Figure 6:**
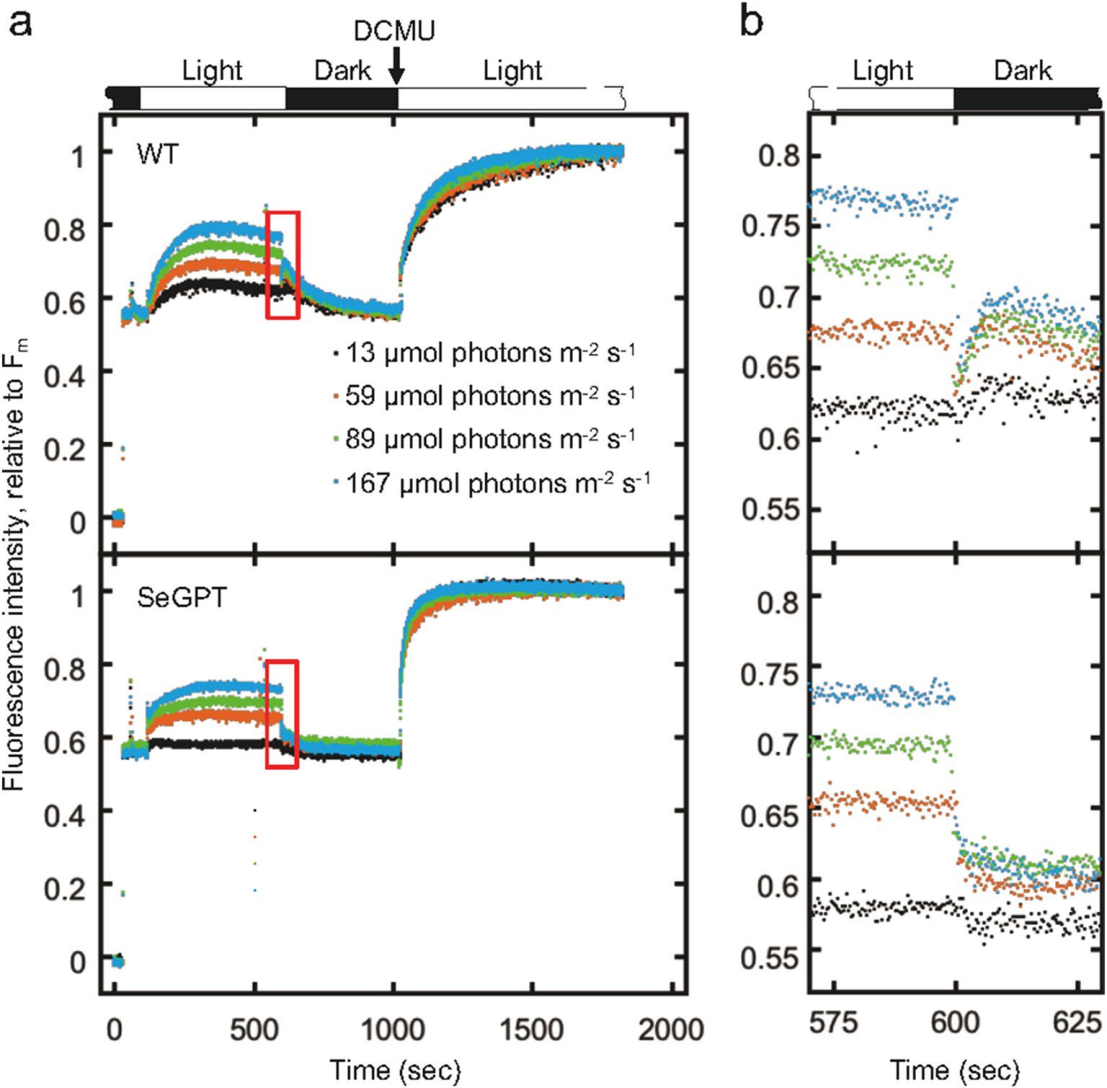
Pulse amplitude–modulated chlorophyll fluorescence measurements. Before fluorescence was measured in the dark (F_0_), cells were dark-acclimated for 10 min. Actinic light at different light intensities was irradiated for 8 min, which was followed by dark relaxation for 7 min. To achieve the maximum fluorescence (F_m_), 10 µM of DCMU was added to the cell suspension and irradiated with actinic light. Representative fluorescence kinetics are shown as relative values normalized to the F_m_. The transient increase in fluorescence immediately after turning off actinic light is shown in the red box (**a**), which is enlarged in (**b**).

Finally, we examined the physiological consequences of the abnormal cell morphologies and altered bioenergetic status in SeGPT. Photosynthetic activities were analyzed using pulse amplitude–modulated chlorophyll fluorescence measurements (Fig. S4a). Figure S4b shows the maximum quantum yield of photosystem II (F_v_/F_m_): no significant difference was observed between SeGPT and the WT (0.369 ± 0.028 and 0.373 ± 0.039, respectively). The effective quantum yields of photosystem II were also examined at various light intensities, but no obvious differences were found (Fig. S4c). However, the transient increase in fluorescence, which was observed in the WT after actinic light was turned off, was almost completely suppressed in SeGPT (Fig. 6a, red boxes). In the WT, this transient increase became more pronounced under stronger actinic light, but no such increase was observed in SeGPT, even under stronger actinic light conditions (Fig. 6b). The fluorescence increase in the WT was diminished by the addition of 2,5-dibromo-3-methyl-6-isopropyl-p-benzoquinone (DBMIB), which is known to bind cytochrome *b*_6_*f* and inhibit plastoquinone oxidation (Fig. S4d). The accumulation of PSII and PSI in the thylakoid membrane was assessed via immunoblotting, and a slight increase in the amount of PSI was observed in SeGPT (Fig. S5, the accumulation was 116 ± 24% for PsbA or 165 ± 18% for PsaA, relative to the WT).

## Discussions

We reported previously that the *Synechococcus* strain SeGPT exhibits increased galactolipid content^24^. It is plausible that heterogeneously expressed MGD1 and DGD2 are no longer subject to the endogenous regulations in SeGPT, and this may affect the total lipid in SeGPT cells. We also demonstrated that SeGPT could grow photoautotrophically, although mild growth retardation was observed^24^. However, the molecular mechanisms underlying these effects were not clarified. In the present study, we identified the causal relationship between the increased lipid content and growth inhibition in SeGPT. Although thylakoid stacking occurred in SeGPT cells, it developed in a more heterogeneous manner than that observed in WT cells. The division of SeGPT cells was impaired, and the cells were longer than those observed in the WT. In SeGPT cells with this abnormal morphology, the levels of ATP and glycogen were increased substantially, suggesting that the bioenergetic status of the cells was altered dramatically, with energy use shifted toward storage rather than cell proliferation. These results indicate that SeGPT cells can be a platform for photosynthetic production of multiple natural or non-natural metabolites. In the current study, we focused on the increase in galactolipids, but we cannot exclude an effect caused by the lack of GlcDG, which should be present in the WT but not in SeGPT due to the displacement of MgdA by MGD1 (Fig. 1a). However, such an effect appears unlikely as no noticeable phenotype was obtained in the SeMPT mutant, in which MGD1 displaced MgdA but no increase in lipids was detected^24^.

In rod-shaped cyanobacteria, thylakoid membranes are formed in regular concentric cylinders aligned along the long axis of the cell^28,29^. Cryo-electron tomography analysis has shown that, during the recovery process after high-light stress in *Synechococcus*, thylakoid membranes develop in a stepwise fashion, i.e., thylakoid lamellae are distributed asymmetrically during the initial stages of thylakoid biosynthesis, after which a more symmetric and regular concentric thylakoid membrane arrangement is established^30^. In several previous studies, small membrane vesicles observed close to the plasma membrane were referred to as a convergence zone, which was suggested to serve a specialized biological function, i.e., coordinating the synthesis of thylakoid proteins and the incorporation of cofactors from the plasma membrane and periplasmic space^30–32^. Although our electron microscope images were a little unclear, the high percentage of unevenly distributed thylakoid membranes in the SeGPT cells indicates that there is an abnormality at the stage of thylakoid membrane spread throughout the cell (Fig. 3).

Because transmission electron microscopy images show a cross-section of a cell at a specific angle, it is difficult to determine the three-dimensional morphology of the cell using such images. Indeed, we were unable to determine the cause of the abnormal cell division, i.e., whether it was caused by abnormal thylakoid membrane formation and/or abnormal cell membrane division. The use of more precise analysis techniques, such as cryo-electron tomography analysis, would be necessary to address this question.

We found previously that DGDG levels in SeGPT cells were increased markedly compared with those in WT cells, and the MGDG/DGDG ratio was reduced to 1.03 in SeGPT cells compared with 1.96 in WT cells^24^. A molecule of MGDG, harboring a small polar head relative to its hydrophobic tail, is cone-shaped and tends to form a structure called hexagonal II, in which the polar heads are assembled inside each other; in contrast, DGDG is cylinder-shaped and forms lamellar structures^33^. The decrease in the MGDG/DGDG ratio by about 50% may have affected the fusion of membrane vesicles and the spread of thylakoid membranes into the cell. Few studies have addressed the relationship between the insufficient development of thylakoid membranes and cell division, which is probably due to the underdeveloped thylakoid membranes reducing photosynthetic activity, inevitably leading to a delay in cell proliferation. In the nonphotosynthetic organism *Escherichia coli*, cell division is inhibited in a transformant with excessive MGDG accumulation, resulting in an elongated cell morphology^34^. Whether the lipid bilayer of the thylakoid membrane is ever continuous with the lipid bilayer of the plasma membrane remains to be elucidated and is a controversial subject^32^. The distribution of lipids, such as DGDG and MGDG, between the thylakoid and plasma membranes in SeGPT is yet to be clarified; thus, further biochemical analysis of SeGPT cells will provide important insights into the relationship between cell division and thylakoid membrane development.

Our chlorophyll fluorescence measurements revealed that photosynthetic linear electron transport is not affected in SeGPT, as indicated by light-response curves of ΦII (Fig. S4c). This result is not contradictory to the previous findings that linear electron transport is severely impaired when galactolipids are reduced^35–37^. Meanwhile, the transient increase observed in the WT immediately after actinic light was turned off was significantly suppressed in SeGPT (Fig. 6a,b). This change in chlorophyll fluorescence indicates the reduction of the plastoquinone pool, as demonstrated by the addition of DBMIB (Fig. S4d), and is considered an indicator of NADPH dehydrogenase 1 complex (NDH-1)-mediated cyclic electron transfer (CET) activity^38–40^. Because it was possible that PSI was significantly reduced in SeGPT (which would also affect the reduction level of the plastoquinone pool), we analyzed the accumulation levels of PSII and PSI, and found a slight increase in PSI in SeGPT compared to the WT (Fig. S5). These results suggest that NDH-1–mediated CET is impaired in SeGPT. We cannot exclude the possibility that CET activity is inhibited by the uneven distribution of thylakoids, which may alter the localization of PSI and NDH-1 on the thylakoid membrane. Further analyses, including native-PAGE of thylakoid proteins followed by immunoblotting with each NDH-1 subunit, may reveal changes in the stability of the complex and composition of the subunits. However, the NDH-1 complex is known to be highly unstable, and the preparation conditions required for the study must be considered carefully^40–42^.

**Figure 7:**
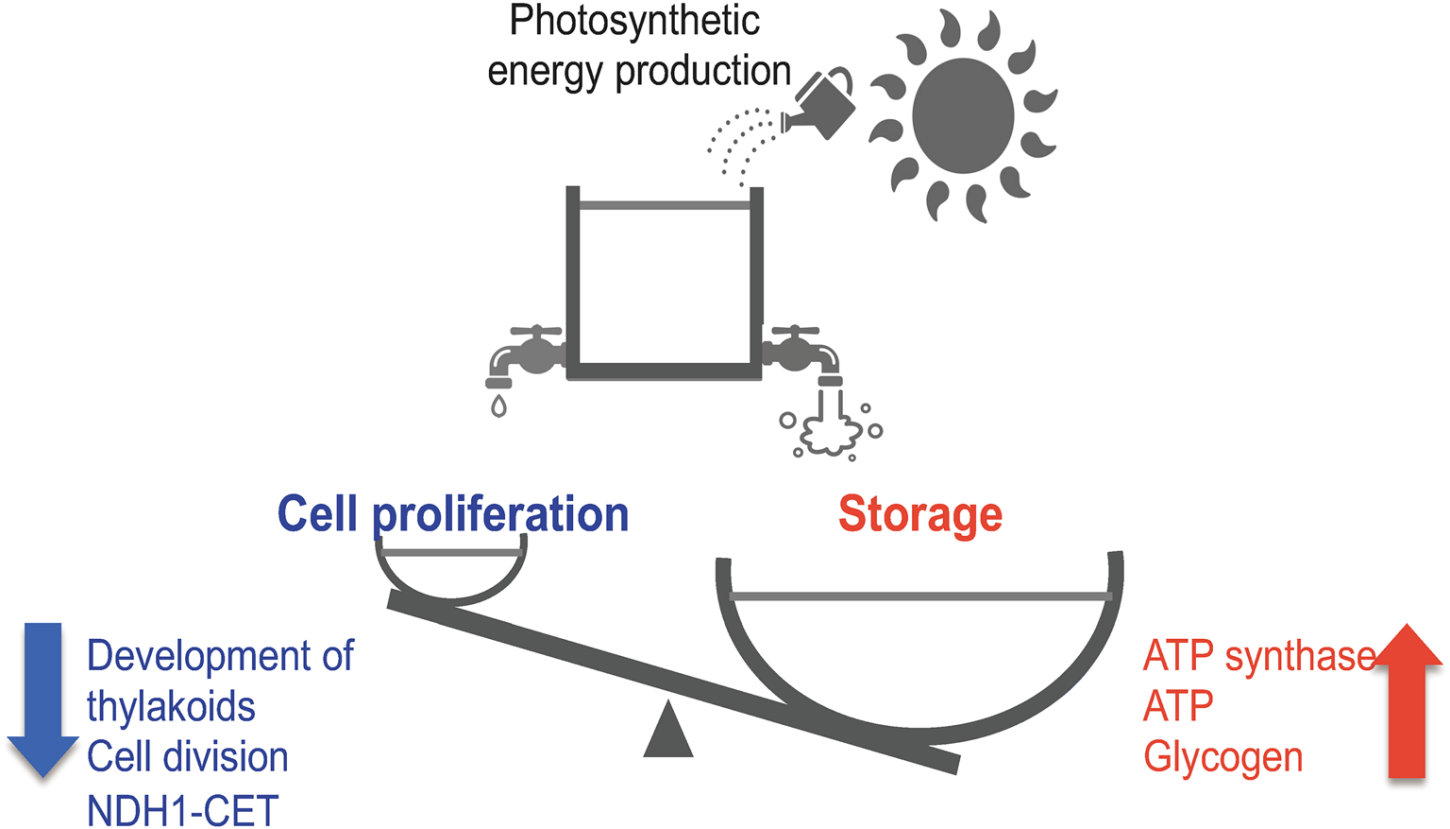
Schematic diagram of the pleiotropic effects observed in SeGPT. A hypothesis of the causal relationships expected from changes in the bioenergetic balance is shown. An increase in galactolipids caused a decrease in the activity of the reactions necessary for cell proliferation, which in turn led to an accumulation of energy storage compounds (see the main text for more details).

In Fig. 7, we present a hypothetical schematic diagram summarizing the pleiotropic effects that occur in SeGPT cells. Following the replacement of the MGDG and DGDG biosynthetic pathways with those from green plants, SeGPT shows increased levels of MGDG and DGDG and a decreased MGDG/DGDG ratio compared with those of the WT. These changes lead to the inhibition of the normal concentric distribution of thylakoid membranes and cell division as well as a reduction in NDH-1–mediated CET activity. We assume that the rate of energy consumption required for cell proliferation is reduced, and the resulting energy surplus is diverted to reactions for energy storage, e.g., ATP and glycogen. For about 10 years, various studies have been conducted to apply cyanobacteria to produce chemicals from carbon dioxide and sunlight^6^. While much of the current research is focused on increasing growth rates, improving photosynthesis and carbon capture, and conferring stress tolerance, cells that accumulate high levels of ATP and glycogen in their cells, may also be promising hosts for material production. In a previous study, Lan and Liao introduced ATP-driven synthesis of acetoacetyl-CoA into *S. elongatus* PCC 7942 and successfully produced 1-butanol by photosynthesis^43^. Meanwhile, Hasunuma et al. reported that overexpression of *flv3* enhances both ATP supply and glycogen biosynthesis, which is a promising approach to biofuel production using cyanobacteria^44^. Furthermore, combining genetic manipulations, such as strategies described above, will be expected to improve synthesis efficiency. Although concerns about the decreased cell proliferation rate in SeGPT remain, we expect that SeGPT will serve as a platform for material production by photosynthetic organisms following further development.

## Methods

### Cell strains and culture conditions

Cells of the WT *S. elongatus* PCC 7942 and its transformant strain SeGPT were obtained and cultured as described previously^24^, including vigorous shaking (120 rpm) under continuous illumination applied using white fluorescent lamps (20–50 µmol photons m^−2^ s^−1^).

### Measurements of chlorophyll content and cell density

For the extraction of chlorophyll, cells were suspended in 100% methanol, after which they were sonicated and centrifuged at 20,000 × *g* for 10 min to precipitate cell debris. Chlorophyll content (µg ml^−1^) was calculated using the following equation:

$${\text{Chlorophyll }}\;{\text{content }} = { 13}.{4 } \times {\text{ A}}_{{{665}}} .$$

Cell density was monitored as the optical density at 750 nm (OD_750_) using a spectrophotometer (UV-1800, Shimadzu) or via direct cell counting (described in the next section).

### Microscopy

Cells at the mid-log phase (5 days after inoculation) or the early-log phase (1 or 2 days after inoculation) were observed under a bright-field microscope (BX-53, Olympus) with an oil-immersion objective lens (PlanApo N 60 × /1.45, Olympus) and photographed using a CMOS camera (STC-5MUSB3, Sentech). Cell colonies on an agar plate were observed under a stereomicroscope (SMZ1000, Nikon) and photographed using a DSLR camera (EOS Kiss X7, Canon). To determine the cell number per OD_750_, a cell-counter plate (Thoma type, WATSON) was applied.

For electron microscopy, cells were chemically fixed as described previously^45^, except that prefixation in 100 mM of potassium phosphate–containing 2.5% glutaraldehyde was extended overnight. Finally, the cells were embedded in an epoxy resin mixture (Epon812 mixture, Nissin EM, Japan), and ultrathin sections were cut with a diamond knife in an ultramicrotome (Leica EM UC7). The sections were placed on copper grids and first stained with 4% samarium chloride aqueous solution for 20 min and then with lead citrate for 5 min. Samples were observed under a transmission electron microscope (JEM-1400Plus, JEOL, Japan) operating at 80 kV. For the evaluation of thylakoid distribution, 22 cells for the WT and 66 cells for SeGPT (22 each for SeGPT1, 2, and 3) were counted.

### Quantification of intracellular ATP/ADP content and the accumulation of F_o_F_1_, PSII, and PSI

The intracellular contents of ATP and ATP + ADP were measured according to a method reported previously^27^. The level of F_o_F_1_ accumulation was assessed using immunoblot analysis with the membrane fraction and antibodies against the β-subunit of F_o_F_1_ as described previously^27^. At the late-log phase, 1 L of cells was harvested via centrifugation, and these cells were flash-frozen in liquid nitrogen and stored at − 80 °C until use. The cells were resuspended in a buffer containing 20 mM of Hepes–KOH (pH 8.0), 10 mM of NaCl, 0.1 mM of MgCl_2_, and 0.1 mM of ATP and broken up via vortexing with zircon beads, after which the homogenate was centrifuged for 10 min at 3000 × *g* and 4 °C to remove cell debris. The supernatant was then centrifuged for 30 min at 125,000 × *g* and 4 °C to precipitate the thylakoid membranes. The chlorophyll concentration of the membrane fraction obtained was then quantified using the methanol extraction method described above and subsequently used for immunoblot analysis. For PSII and PSI analyses, whole-cell extracts and the antibodies against PsbA and PsaA were used (AS06 124A and AS06 172; supplied by Agrisera, Sweden).

### Quantification of intracellular glycogen content

Cells grown under continuous light conditions were harvested, washed twice with distilled water, and resuspended in 500 µl of 3.5% (v/v) sulfuric acid, followed by incubation at 100 °C for 120 min. Glycogen was quantified using a LabAssay Glucose Kit (Wako, Japan) as described previously^27^.

### Photosynthetic activity measurements

Chlorophyll fluorescence was measured using a Dual-PAM 100 Fluorescence Measuring System (Walz, Germany). Prior to the measurements, *Synechococcus* cells were adjusted to OD_750_ = 0.3. The minimum chlorophyll fluorescence (F_0_) was measured under dark conditions. Actinic light treatment was then applied to obtain the fluorescence parameters, steady-state fluorescence (F), and maximum fluorescence in the light (F_m_’). Subsequently, the actinic light was turned off for 7 min, after which 10 µM of DCMU was added. The maximum chlorophyll fluorescence (F_m_) was then determined after reirradiation of the actinic light. The photosynthetic parameter was calculated using the following Eq.^46^:

$${\text{F}}_{{\text{v}}} /{\text{F}}_{{\text{m}}} = \, \left( {{\text{F}}_{{\text{m}}} - {\text{ F}}_{0} } \right)/{\text{F}}_{{\text{m}}} ;$$

$$\Phi {\text{II }} = \left( {{\text{F}}_{{\text{m}}}{\text{'}} - {\text{F}}} \right)/{\text{F}}_{{\text{m}}} .$$

## Supplementary Information

Supplementary Information.

## Data Availability

All data are contained within the article and can be shared upon request (correspondence: thisabor@res.titech.ac.jp and awai.koichiro@shizuoka.ac.jp).
