# Author Correction: Changes in intracellular energetic and metabolite states due to increased galactolipid levels in *Synechococcus elongatus* PCC 7942

**DOI:** 10.1038/s41598-023-29745-z

**Published:** 2023-02-15

**Authors:** Kumiko Kondo, Rina Yoshimi, Egi Tritya Apdila, Ken-ichi Wakabayashi, Koichiro Awai, Toru Hisabori

**Affiliations:** 1 Laboratory for Chemistry and Life Science, Institute of Innovative Research, Tokyo Institute of Technology, Nagatsuta 4259-R1-8, Midori-Ku, Yokohama, 226-8503 Japan; 2 School of Life Science and Technology, Tokyo Institute of Technology, Nagatsuta 4259, Midori-Ku, Yokohama, 226-8503 Japan; 3 Department of Biological Science, Faculty of Science, Shizuoka University, Suruga-Ku, Shizuoka, 422-8529 Japan

Correction to: *Scientific Reports* https://doi.org/10.1038/s41598-022-26760-4, published online 05 January 2023

The original version of this Article contained an error in Reference 37, which was incorrectly given as:

Wu, W. *et al.* Monogalactosyldiacylglycerol deficiency in tobacco inhibits the cytochrome b6f-mediated intersystem electron transport process and affects the photostability of the photosystem II apparatus. *Biochim Biophys. Acta* **709–722**, 2013. https://doi.org/10.1016/j.bbabio.2013.02.013 (1827).

The correct reference is listed below:

Wu, W. *et al.* Monogalactosyldiacylglycerol deficiency in tobacco inhibits the cytochrome b6f-mediated intersystem electron transport process and affects the photostability of the photosystem II apparatus. *Biochim. Biophys. Acta* **1827**, 709–722. https://doi.org/10.1016/j.bbabio.2013.02.013 (2013).

The original Article has been corrected.

