# Supplementary material for: Changes in intracellular energetic and metabolite states due to increased galactolipid levels in Synechococcus elongatus PCC 7942
Source: Sci Rep. 2023 Jan 5;13:259. doi: 10.1038/s41598-022-26760-4 (PMC9816115; doi:10.1038/s41598-022-26760-4)
Supplement: Supplementary file 1 — Supplementary Information. [file 41598_2022_26760_MOESM1_ESM.pdf]

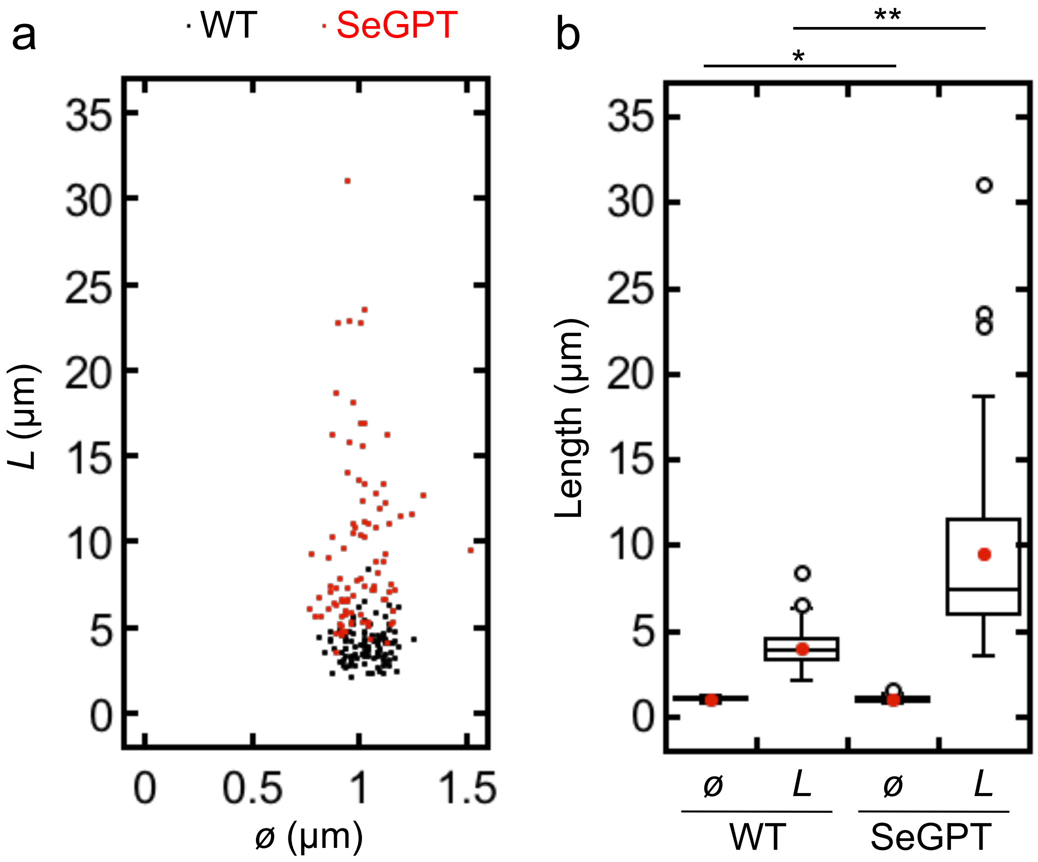

Fig. S1. Measurements of cell size at the early-log phase (1 or 2 days after inoculation). Cell length *(L)* and diameter (*ø*) were compared between WT and SeGPT. The results are presented in dot plots *(a)* and box plots *(b).* Black and red dots indicate WT and SeGPT, respectively *(a).* Red circles indicate the averages (*b,* n = 103 for WT, and 94 for SeGPT). The asterisks indicate statistical significance (*P < 0.001, **P < 0.1^12, Welch's t-test).

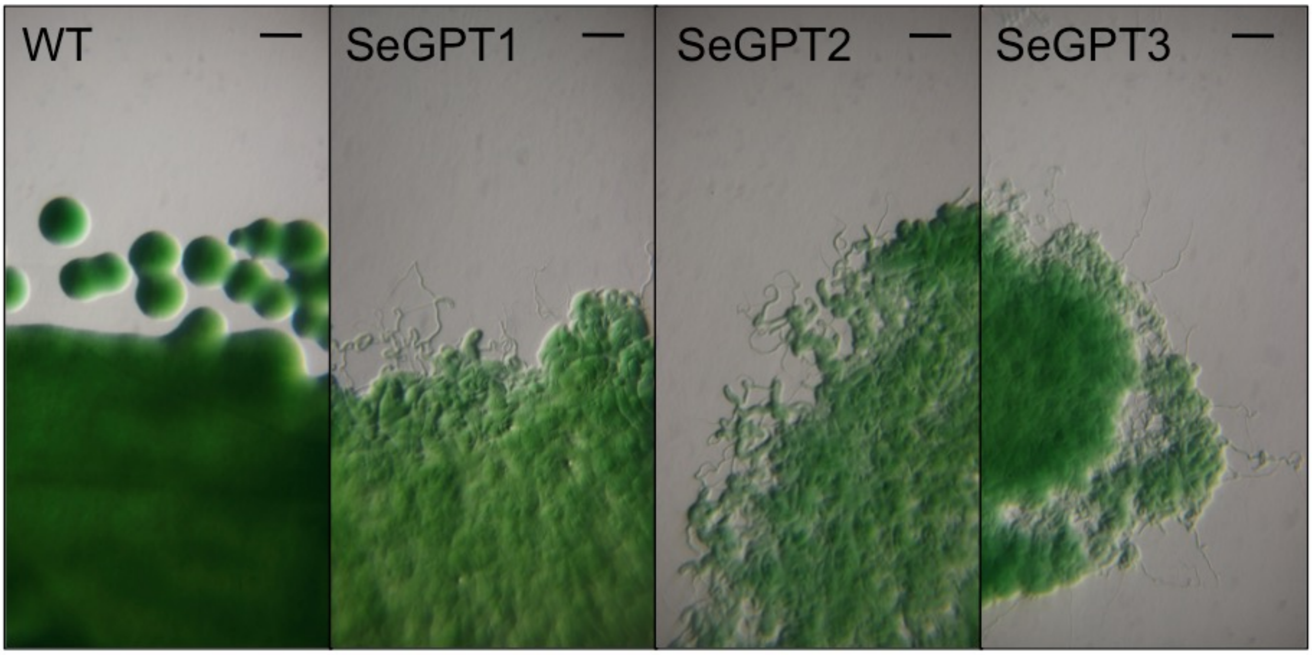

Fig. S2. The margins of the colonies on BG11-agar in plate cultures of WT and SeGPT. Images were taken by using stereomicroscope. The formation of the margins were compared with WT and SeGPT mutant strains (SeGPT1, 2, and 3). SeGPT1 corresponds to SeGPT in main text. Bars indicate 200 μm.

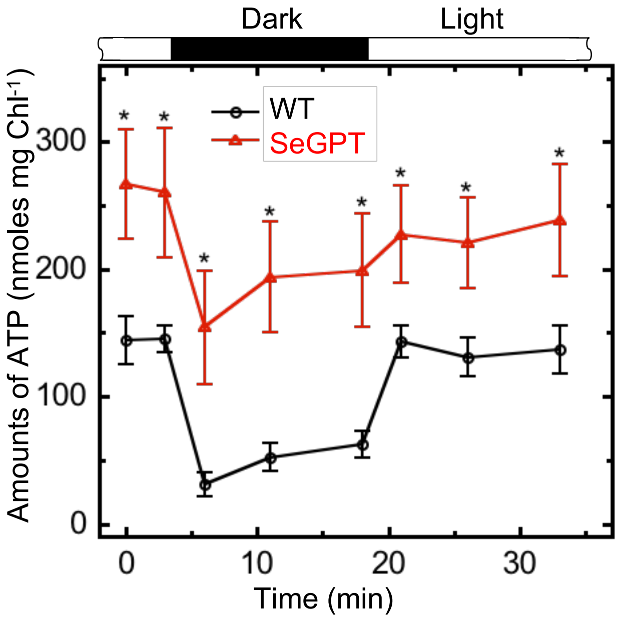

Fig. S3. Short-term kinetics of intracellular ATP levels during a light–dark–light transition. Cells grown under continuous illumination were treated with dark incubation for 15 min, followed by illumination at 30 µmol photons m^{-2} s^{-1}. Cells were withdrawn at the indicated time points and ATP content was quantified as described in Materials and Methods. The results of 8 independent experiments were averaged (mean ± SD). Asterisks indicate statistical significance ($P < 0.05$, Welch's t-test).

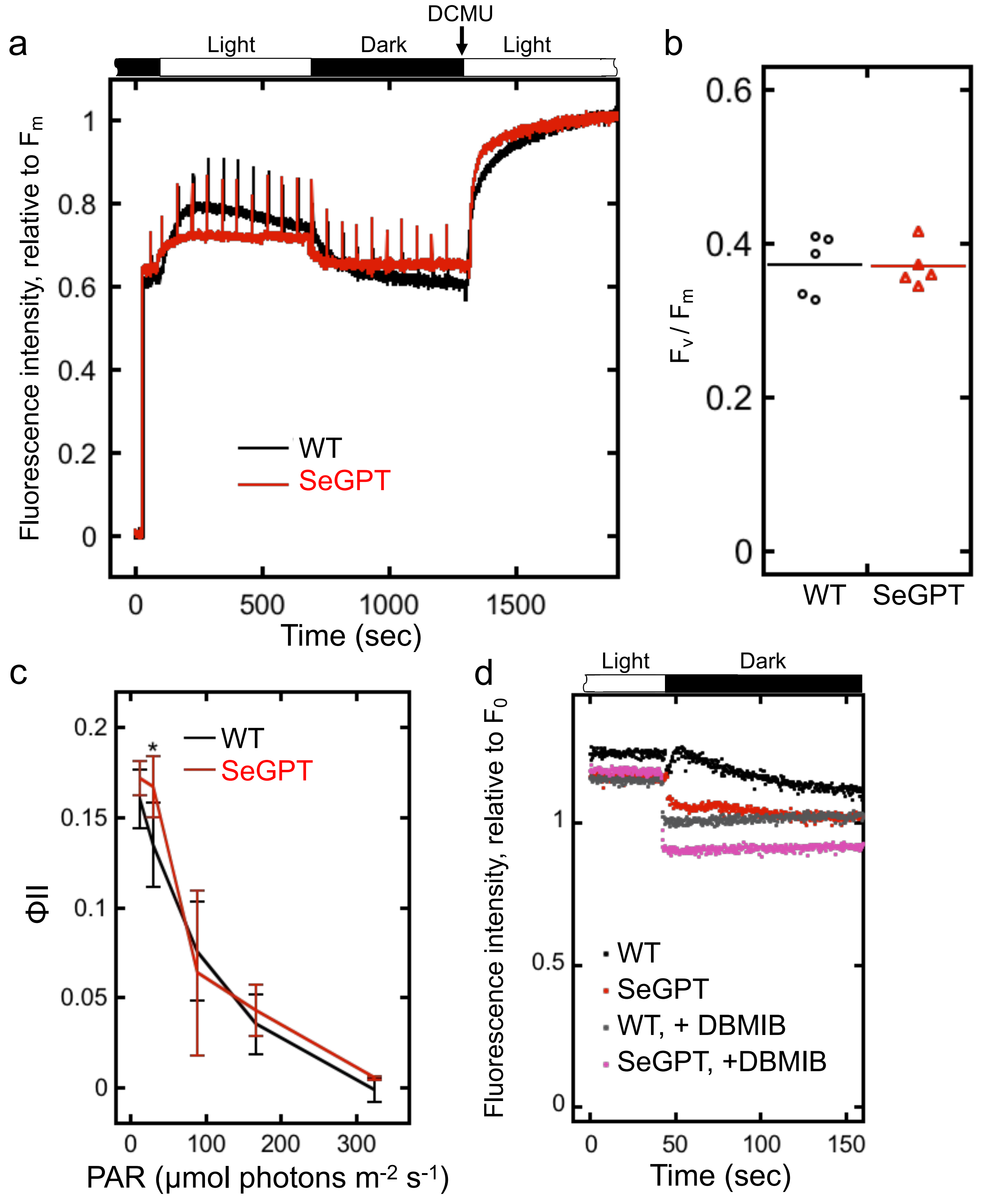

Fig. S4. Pulse-amplitude-modulated chlorophyll fluorescence measurements of WT and SeGPT. (*a*) Chlorophyll fluorescence kinetics of WT (black line) and SeGPT (red line). Prior to the measurements, cells were dark-acclimated for 10 min. Actinic light treatment (30 μmol photons m^{-2} s^{-1}) was started after dark relaxation for 30 sec. To get the maximum fluorescence (F_m), 10 μM DCMU was added to the cell suspension and irradiated with actinic light. Representative fluorescence kinetics are presented as relative values normalized to F_m. (*b*) The values of F_v/F_m were shown as dot plots. Bars indicate the average (n = 5). *(c)* Light-response curves of PSII quantum yield (ΦII) kinetics of WT (black line) and SeGPT (red line). Each ΦII was determined from the values obtained when exposed to actinic light for 10 minutes. PAR, Photosynthetic active radiation. The results of 3-5 independent experiments were averaged (mean ± SD). The asterisk indicates statistical significance (*P* < 0.05, Welch's t-test). *(d)* Transient increase of fluorescence observed in WT (black) was diminished by the addition of 5 mM DBMIB (gray) or in SeGPT (red and pink). Actinic light intensity was 59 μmol photons m^{-2} s^{-1}. Data are presented as the relative values normalized to F_0.

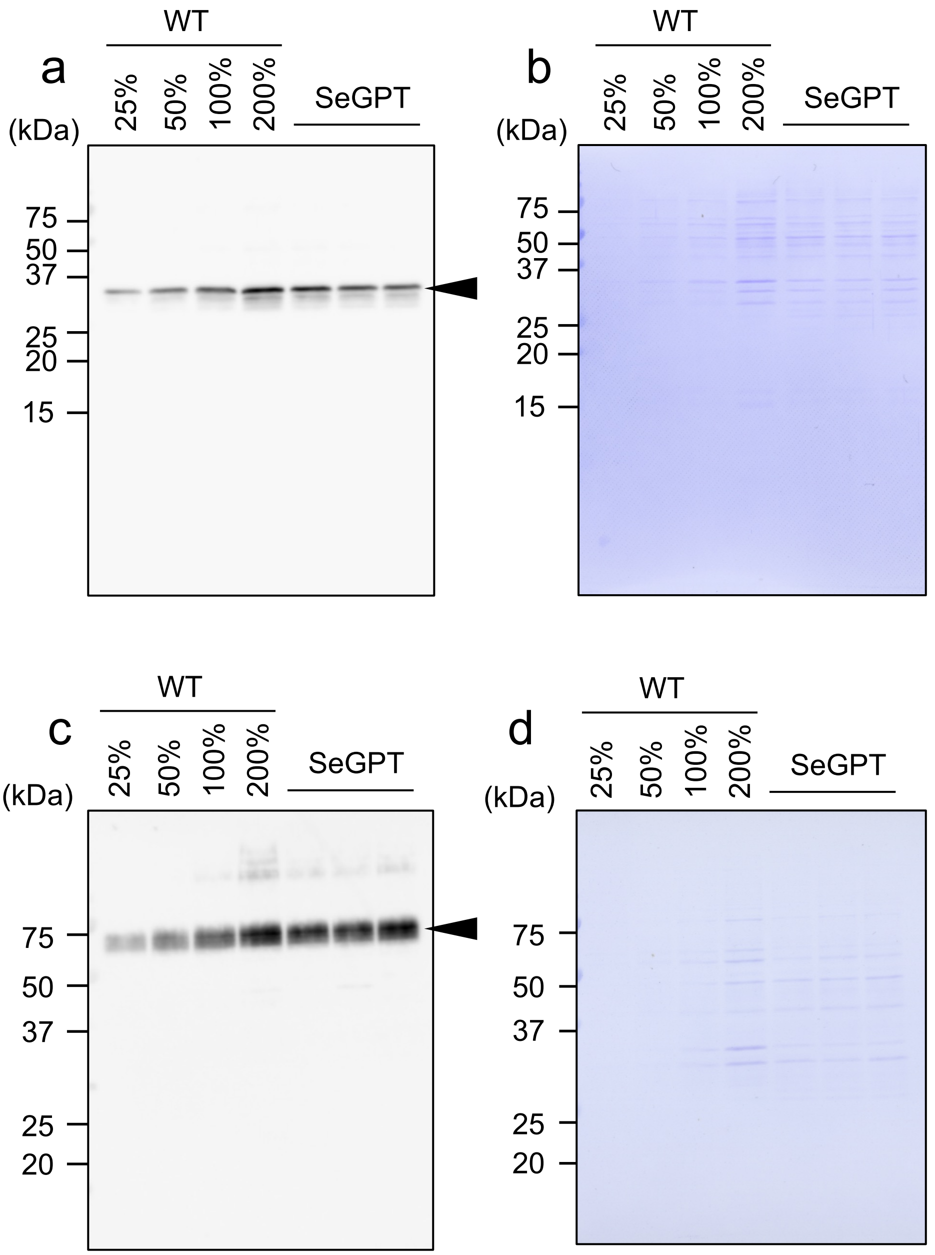

Fig. S5. Immunoblot analyses of PsbA (*a*) or PsaA (*c*) and CBB-stained membranes (*b*, *d*). Immunoblotting was performed using antibodies against PsbA (*a, b*) or PsaA (*c, d*). Cell crude extracts equivalent to 40 ng chlorophyll were loaded for 100% WT and SeGPT. Three extracts from independent experiments were loaded for SeGPT. Representative images from more than three independent experiments are shown. Full-length images are shown in Fig. S6.

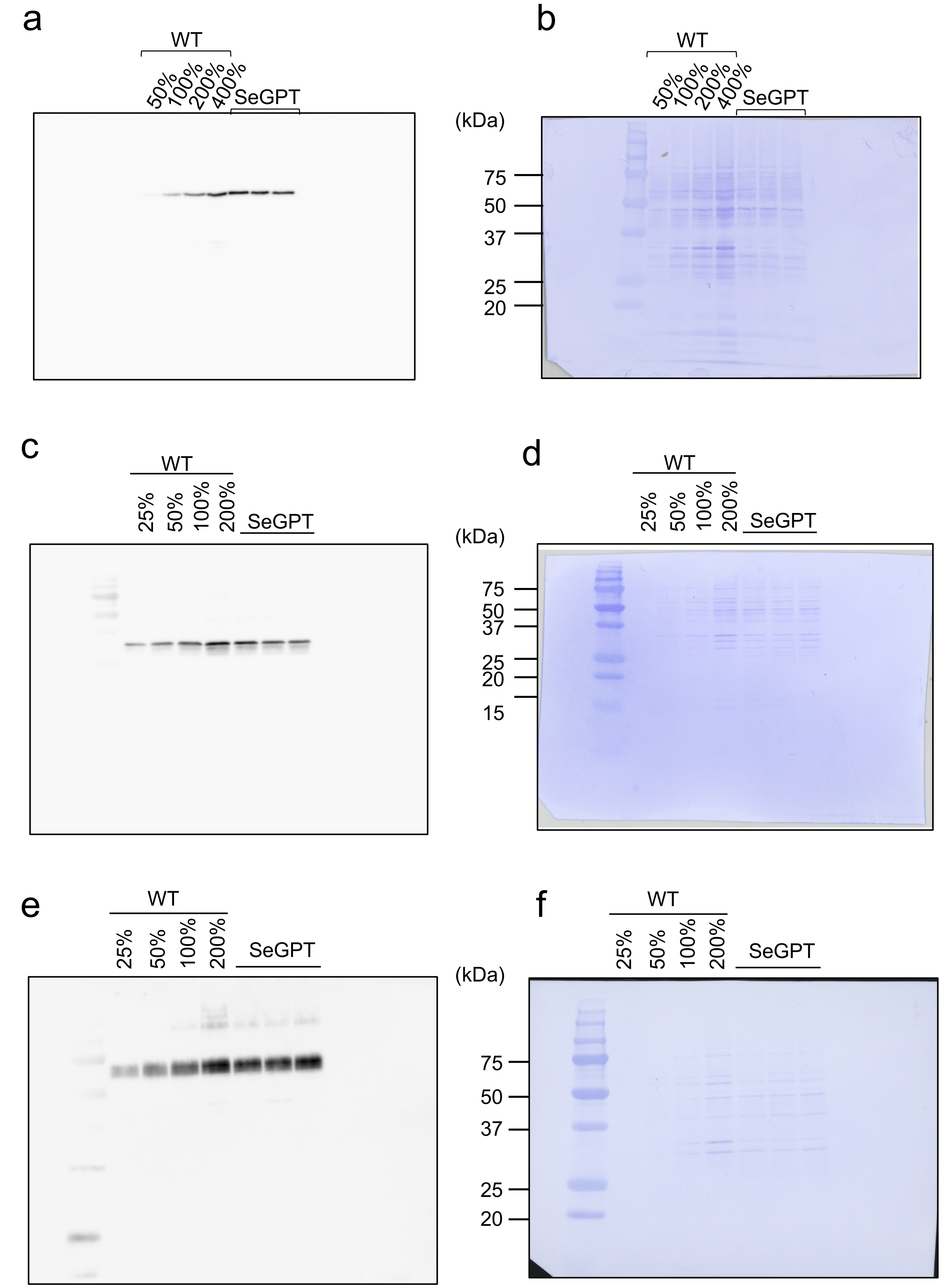

Fig. S6. Full-length images of membranes shown in Fig. 4 and S5. Immunoblot analyses of F_oF_1-β (*a*), PsbA (*c*) or PsaA (*e*) and CBB-stained membranes (*b*, *d, f*).
